# Regulation of Internet-based Genetic Testing: Challenges for Australia and Other Jurisdictions

**DOI:** 10.3389/fpubh.2018.00024

**Published:** 2018-02-15

**Authors:** Jane Tiller, Paul Lacaze

**Affiliations:** ^1^Public Health Genomics, Department of Epidemiology and Preventive Medicine, School of Public Health and Preventive Medicine, Monash University, Melbourne, VIC, Australia

**Keywords:** direct-to-consumer genetic testing, regulation, public health genomics, Australia, Therapeutic Goods Administration

## Abstract

The Internet currently enables unprecedented ease of access for direct-to-consumer (DTC) genetic testing, with saliva collection kits posted directly to consumer homes from anywhere in the world. This poses new challenges for local jurisdictions in regulating genetic testing, traditionally a tightly-regulated industry. Some Internet-based genetic tests have the capacity to cause significant confusion or harm to consumers who are unaware of the risks or potential variability in quality. The emergence of some online products of questionable content, unsupported by adequate scientific evidence, is a cause for concern. Proliferation of such products in the absence of regulation has the potential to damage public trust in accredited and established clinical genetic testing during a critical period of evidence generation for genomics. Here, we explore the challenges arising from the emergence of Internet-based DTC genetic testing. In particular, there are challenges in regulating unaccredited or potentially harmful Internet-based DTC genetic testing products. In Australia, challenges exist for the Therapeutic Goods Administration, which oversees regulation of the genetic testing sector. Concerns and challenges faced in Australia are likely to reflect those of other comparable non-US jurisdictions. Here, we summarize current Australian regulation, highlight concerns, and offer recommendations on how Australia and other comparable jurisdictions might be more proactive in addressing this emerging public health issue.

## Introduction

**Figure 1:**
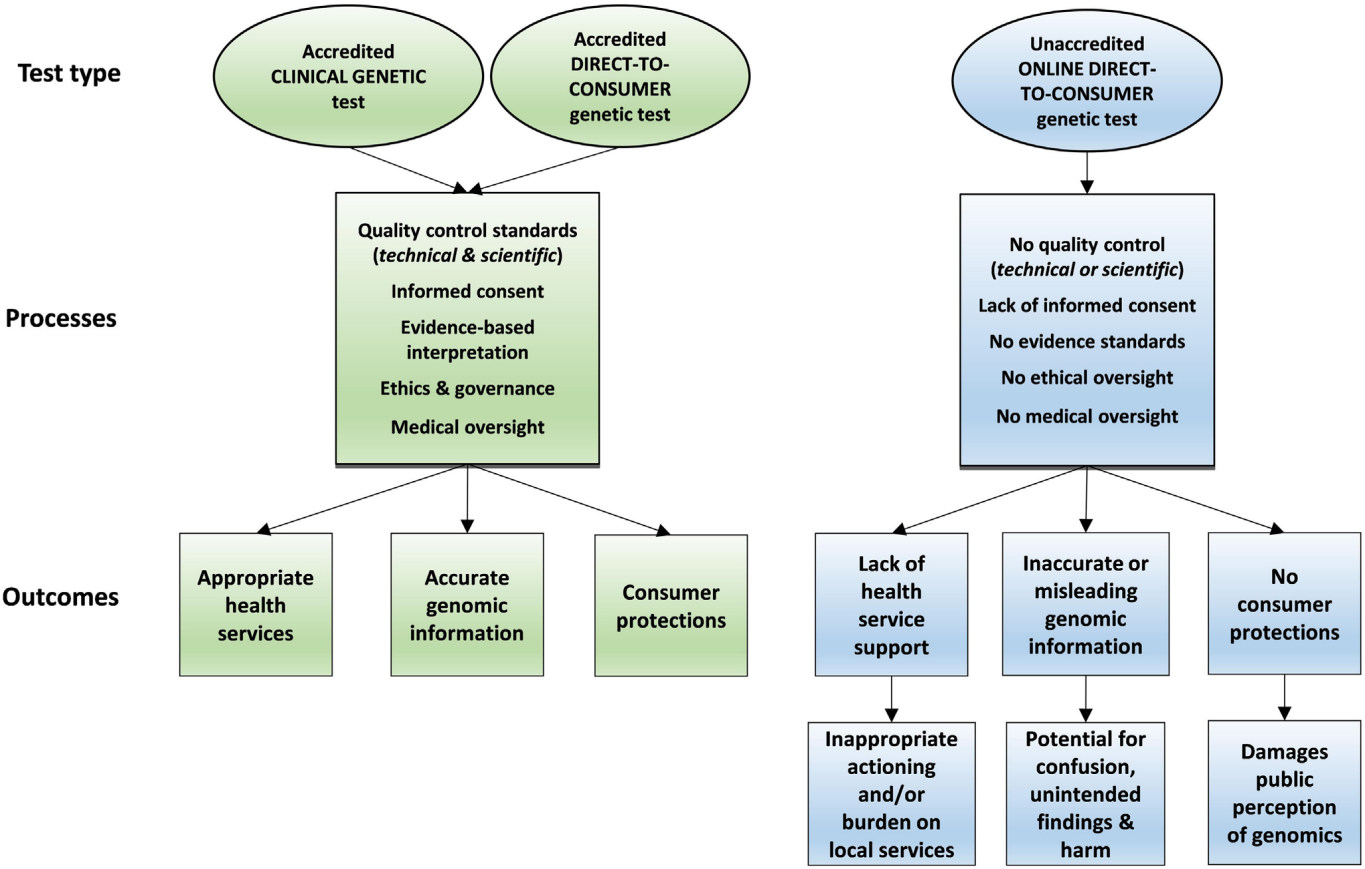
Processes and outcomes for accredited versus non-accredited genetic testing pathways.

A direct-to-consumer (DTC) genetic test is any DNA test for a medical or non-medical trait that provides interpretation or communication of test results directly to a consumer, rather than via a health professional. DTC genetic tests are often accessed *via* the Internet without the need for a medical referral, outside of the health system. Sample collection kits can be posted directly to the consumer without involvement from any health professional. Internet-based DTC genetic tests vary in price, quality, and genetic content measured, ranging from “recreational” testing (1) to return of medical disease risk information (2). Online DTC genetic tests are growing in popularity due to various consumer motivations, many of which are not necessarily medical in nature (2, 3). There are several potential harms and consequences of poorly regulated Internet-based DTC testing, which have been well documented (4–6) and are summarized in Figure 1.

Online DTC genetic tests are generally delivered in the absence of genetic counseling or medical oversight. Some consumers with DTC test results are now looking to general practitioners or clinical genetic services for assistance with interpretation or management of DTC genetic findings, posing an emerging challenge for the medical community (6, 7).

Many online DTC genetic tests originate in the USA, where the Food and Drug Administration (FDA) has ongoing challenges in maintaining regulatory oversight (8). Online DTC tests originating from the USA under FDA approval do not necessarily obtain country-specific approval elsewhere in non-US jurisdictions. However, many are still available and accessible *via* the Internet from any country, essentially by-passing local testing regulations in non-US countries. Some online DTC tests, if sold locally in non-US jurisdictions, would be in violation of local guidelines for genetic testing. However, direct access *via* a global online marketplace creates challenges for non-US authorities in enforcing local regulations on Internet-based products.

How local jurisdictions, such as Australia, the UK, and Europe, should approach regulation and quality control of Internet-based genetic testing is uncertain (9–12). The immediate availability and direct nature of access pose new challenges. Although difficult, many of these challenges are not necessarily unique to the field of genetic testing and have been mirrored in other regulated industries recently disrupted by the emergence of a global online marketplace, such as the online prescription drug sector (13).

## Current Regulation of Genetic Testing in Australia

Under current Australian regulation, there is a strict regulatory regime governing the registration and provision of human genetic tests offered by Australian companies (14–17). Furthermore, laboratories which carry out genetic testing must be accredited for technical competencies by the National Association of Testing Authorities (18). These standards mandate a level of quality control for genetic testing services in Australia. However, compliance with these standards makes it challenging, and relatively expensive, for Australian companies to provide price-competitive DTC testing services compared with offshore DTC companies. Such offshore companies can access Australian consumers *via* the Internet, but are not subject to any Australian regulation.

Consumers may have difficulty distinguishing between locally accredited Australian products and unaccredited, offshore products marketed online. The inability of local authorities such as the Therapeutic Goods Administration (TGA) to regulate online DTC genetic testing and advertising leads to a multitude of regulatory, medical, and ethical concerns, which are set out below and summarized in Table 1. In addition, Australian regulation explicitly allows consumers to access non-accredited overseas tests through a self-importation exemption [(14), Reg 7.1 and Schedule 4].

**Table 1:** Concerns with unaccredited online direct-to-consumer (DTC) genetic testing.

Regulation/quality	Challenging for local authorities to regulate online products
	No technical standards for quality control
	No scientific standards for evidence of significance or actionability

Medical	Return of actionable genetic findings without medical oversight
	DTC customers seek interpretation from local health services
	Potentially damaging to the reputation of medical genomics

Ethical	Return of actionable genetic findings without genetic counseling
	Disclosure of risk variants for non-treatable conditions
	Erosion of informed consent
	Recreational intent versus unintended genetic findings

Privacy	DTC companies retain consumer data and DNA samples
	Access to genetic data by third parties, without consumer consent

## Concerns with Unaccredited Internet-Based DTC Genetic Tests

### Regulation/Quality

Although stringent standards apply to genetic testing conducted in Australia, the TGA and other regulators are not empowered to prevent access to or regulate the quality of Internet-based DTC genetic tests conducted overseas. Similar issues are faced by other international regulators (9), with issues reported such as difficulties determining whether DTC samples were being processed locally or sent overseas (11). Given the challenges of genomic literacy in the general population (19, 20), many consumers may not be aware of the quality of online genetic tests. Thus, consumers are vulnerable to online marketing by overseas companies, especially for some of the more questionable products generally opposed by the scientific and medical community (10, 21).

### Medical Issues

There is evidence consumers of Internet-based genetic tests are increasingly seeking the advice of general practitioners or clinical genetics services for interpretation of results (22). This risks placing an increased burden on existing local health services, which are often publicly funded with limited resources. Funding of additional services to accommodate a growing influx of DTC consumers may not be sustainable in Australia and other comparable nations (23), particularly when results can be ambiguous, uncertain, or confusing, and often identified in individuals not at genuinely increased risk of disease. With some Internet-based DTC companies returning significant genetic risk information of medical and psychological gravity, such as variants in the BRCA genes, without any genetic counseling or medical support, there is also scope for potential harm (24) and/or inadequate care for those who need it.

Furthermore, consumers may have difficulty in distinguishing between established locally accredited clinical genetic testing services (meeting high standards of quality control), versus cheaper online options not subject to the same quality measures. This has the potential to confuse consumers and may compromise long-standing efforts of local genetic services (25).

### Ethical Issues

Consumers purchasing DTC genetic tests may be motivated by curiosity, ancestry, or recreational motivations rather than medical reasons. However, they may uncover serious medical risk factors, non-paternity, or other unexpected genetic information in the process of testing, without having considered the implications beforehand (5, 26). In addition, some online tools can now be used to analyze raw genetic data from non-medical DTC tests (such as ancestry tests), to generate interpretations of medical risk. This means individuals can now access medical risk information from raw genetic data online, without any regulation, quality control, or medical oversight after undertaking an ancestry test. This opens up the potential for incorrect interpretation as well as the return of genuinely medically significant risk information without informed consent, genetic counseling, or medical oversight (27).

Genetics services providing clinical testing in Australia follow international guidelines regarding the evidence required to substantiate medical risk information before it is provided to the consumer (28). Model guidelines have also been developed for the evaluation of genetic tests (29), but online DTC companies can provide medical risk information to consumers without fulfilling these evidence requirements (30). Informed consent for Internet-based DTC products does not meet traditional clinical genetic standards, with most DTC companies currently not providing pre- or post-test genetic counseling or medical support (10).

Some DTC tests return genetic risk information for untreatable conditions prior to symptom onset, such as the APOEe4 risk allele of Alzheimer’s disease (31). Although some studies have shown such results can be used by at-risk individuals to plan ahead (3), direct provision of this information without access to genetic counseling or medical oversight is generally not standard practice in the clinical genetics community, and is considered by many to be unethical (32). Media reports have detailed anecdotes of individuals who have unexpectedly received risk information for Alzheimer’s disease through DTC testing and experienced distress as a result (33).

### Privacy Issues

The increasing number of consumers providing DNA samples to online companies also raises concerns around the privacy of genetic data. Recent studies have shown that many online DTC companies do not consistently meet international guidelines regarding data use and privacy (34), and consumers’ expectations around privacy and use of their genetic data can be inconsistent with companies’ practices (35). Many online DTC companies retain DNA samples for subsequent use, including research, with potentially ambiguous consumer information about the use and storage of DNA samples (36). Furthermore, it has been suggested that online DTC companies are selling access to their databases of genetic information to third parties, or providing samples for research purposes, potentially without the knowledge or consent of consumers who provided the data (34, 35).

### Future Considerations and Recommendations

Given the growing fascination with genetic testing, it is inevitable consumers will continue to seek Internet-based DTC products. The demand for cheap, Internet-based DTC genetic testing may also be fueled by the lack of access to, and cost of, locally accredited clinical genetic testing options in some countries, especially those with publicly funded health systems (37).

There is currently no international association tasked with regulating the online DTC market. The Global Alliance for Genomics and Health (38) is developing standards for the sharing of genomic data, but does not have regulatory powers. The limited amount of public funding allocated for clinical genetic testing in most countries, combined with the increased demand for clinical genetic testing, means many individuals who do not qualify for publicly funded testing under current guidelines may seek alternative, low-cost ways of obtaining genetic information directly.

Unless governments take steps to inform consumers of the dangers of some online DTC genetic testing products, or provide alternative testing pathways, it is likely that consumers will continually have difficulty distinguishing between quality (locally accredited) and non-quality (unaccredited) online products. Many consumers may choose low-priced, low-quality tests and therefore be vulnerable to many of the medical, ethical, and privacy concerns. The potential for confusion, unexpected outcomes, and harm will increase and could threaten the public perception of genomics at a critical time. It is vital that public faith and engagement are safeguarded during the ongoing period of evidence generation for implementation of genomic medicine.

In the future, the concept of governments or public health systems providing access to universal, population-wide genomic screening for disease prevention needs to be considered. This would provide an alternative testing pathway to unregulated Internet-based DTC testing accessed through the private sector. It would ensure stronger quality control, appropriate informed consent, and implementation of evidence-based prevention following national screening principles (39). A recommendation in this regard is set out below. If publicly funded screening is not implemented, it is likely Australia and other jurisdictions will continue to see consumers gravitate toward cheap, Internet-based genetic testing options, especially when genomic literacy remains low.

We recommend the Australian government and comparable jurisdictions take the following steps:

1. Promote education of the public regarding DTC genetic testing, including publicizing warnings in prominent and widely accessed media about risks of unaccredited online DTC genetic testing products.
2. Publicly endorse any local or international companies whose genetic tests meet local accreditation standards, though an easily recognizable accreditation icon, so that consumers can readily identify valid and approved tests.
3. Amend current regulations so that personal importation of unaccredited genetic tests is not sanctioned.
4. Prohibit Internet advertising of non-accredited offshore tests and engage with overseas regulators regarding strategies for regulating advertising of, and access to, online tests.
5. Implement compulsory guidelines requiring the application of evidence requirements for interpretation of genetic tests before the return of results to consumers.
6. Consider a proof-of-concept study to pilot the development of a low-cost, publicly funded, population genomic screening program for young adults, linked with the health system, accompanied by education, focused on the delivery of evidence-based, medically useful risk information for those who seek it.

The implementation of these recommendations would require significant allocation of resources by the government, both toward regulation of online tests and steps toward building a health system capable of undertaking population genomic screening, including scaling of genetic counseling and other medical services. Significant feasibility studies and health-economic modeling will be required before this can become a reality.

The future landscape of genetic testing in countries with strong public health systems, such as Australia, remains uncertain. Many individuals will continue to seek DTC testing *via* the online marketplace regardless, especially for recreational purposes such as ancestry testing, which have limited potential for harm. However, for medical risk information, there are more complexities to consider.

The prospect of a national genomic screening program in Australia to identify actionable genetic risk in consenting adults could be considered. This could potentially identify preventable disease risk early, which if linked to public health system services, could enable closer and more appropriate medical, scientific, and ethical oversight for mainstreaming of genomic testing. A public health screening strategy would ensure those genuinely at-risk are identified and offered appropriate clinical genetic services when needed. Under this model, only established actionable genetic findings, supported by clinical guidelines and standard-of-care for preventable disease, would be disclosed (meaning most individuals would not be receiving results). This may make interpretation of genomic results and subsequent medical risk assessments more achievable.

Screening could be accompanied by national education and genomic literacy programs. These efforts may deter people from seeking unaccredited DTC testing products online for medical disease risk assessment and encourage appropriate management for those genuinely at risk. The prospect of genomic population screening linked to a public health system would require significant bolstering of Australian clinical genetic services, far beyond the current scope. This would need substantive increases in public funding and infrastructure. Steps in this direction will need to be considered as the wave of consumers turning to DTC testing will continue to rise in coming years.

The Internet, combined with an increasing public fascination in genomics, is currently resulting in an unprecedented access to genetic testing. This will continue to rise and present new challenges for nations in regulating testing and interpretation services. It is likely a pro-active and forward-thinking approach to regulation will be required.

## Author Contributions

JT drafted the initial manuscript. JT and PL contributed to each draft of the manuscript.

## Conflict of Interest Statement

The authors declare that the research was conducted in the absence of any commercial or financial relationships that could be construed as a potential conflict of interest. JT provides legal consulting services to genetic testing companies.
